# Genetic polymorphisms (*FTO* rs9939609 and *TMEM18* rs6548238), adipokines (leptin and adiponectin) and adiposity in children and adolescents with asthma

**DOI:** 10.1016/j.jped.2024.07.006

**Published:** 2024-08-16

**Authors:** Marta Evangelho Machado, Luis C. Porto, Jeane S. Nogueira, Clemax C. Sant´Anna, José R. Lapa e Silva

**Affiliations:** a Universidade Federal do Rio de Janeiro (UFRJ), Programa de Pós-graduação em Clínica Médica (PPGCM - HU), Rio de Janeiro, RJ, Brazil; b Fundação Técnico-Educacional Souza Marques, Departamento de Pediatria – Pólo Itanhangá, Rio de Janeiro, RJ, Brazil; c Universidade do Estado do Rio de Janeiro (UERJ), Laboratório de Histocompatibilidade e Criopreservação (HLA - PPC), Rio de Janeiro, RJ, Brazil

**Keywords:** Single nucleotide polymorphism, Gene, Allele, Obesity, Overweight, Asthma

## Abstract

**Objective:**

To describe independent factors related to the interaction of *FTO* rs9939609, *TMEM*18 rs6548238, leptin, and adiponectin in children/adolescents with asthma, under the influence of obesity.

**Methods:**

The authors performed a cross-sectional study with 57 children/adolescents, ages 8–19 years, at a tertiary hospital, from 2017 to 2018. Participants were classified by nutritional status, performed spirometry with a bronchodilator test and completed an asthma questionnaire, higher scores indicated more asthma symptoms. Two asthma groups were formed: Group 1(G1)-normal-weight; Group 2(G2)-overweight/obese. Serum was collected for adipokines (*n* = 32) and genetic polymorphisms (*n* = 53) dosages.

**Results:**

Age and body mass index (BMI) correlated directly in normal-weight (*p* = 0.009) and obese participants (*p* = 0.004). Girls reported more asthma complaints (*p* = 0.044). Participants with negative bronchodilator responses presented lower BMI (14.55–17.16) than responders (19.4–26.84) (*p* = 0.049). Leptin dosages are related directly to BMI (5,34–40 ng/ml in obese × 0,54–42 ng/ml in nonobese) (*p* = 0.003). Levels were high in girls (4.78–17.55 µg/ml) (*p* = 0.029) and low in nonobese boys (0.54–6.92 µg/ml) (*p* = 0.006). In obese, low leptin levels (< 10 ng/ml) were found in small airway dysfunction carriers (*p* = 0.025); elevated adiponectin (> 5 µg/ml) correlated with FEV1/FVC > 80 % (*p* = 0.035) and positive bronchodilator tests (8.84–13 µg/ml) (*p* = 0.039); and *FTO* A allele correlated with low adiponectin 0–8.84 µg/ml (*p* = 0.021) and low FEV1/FVC (46 %-88 %) (*p* = 0.023).

**Conclusion:**

BMI correlated directly with age and leptin levels. Obese participants presented high serum levels of leptin and *FTO* A allele correlated with low FEV1/FVC. Larger cohorts are necessary for better elucidation of the role of adipokines and polymorphisms in the pathophysiology of asthma and obesity.

## Introduction

To date, the pathogenic relationship between asthma and obesity in children has not been fully understood. The knowledge about the participation of genes like Fat mass and obesity-associated **(**FTO) and Transmembrane protein **(***TMEM)* 18 and adipokines like leptin and adiponectin, in asthma, under the influence of obesity, is still scarce.

Adiposity is associated with high adipokines serum levels and, in asthmatic children and adolescents, directly associated with leptin concentration.^1^ Leptin increases Interferon γ–mediated responses, activates mast cells and transcription factors, promotes leukotrienes production, increases immunoglobulin E secretion,^2^^,^^3^ and Th1 cytokines, suppresses the production of Th2 cytokines and increases the release of vascular growth factors, stimulating vascular permeability, findings of inflammatory lung diseases. Negative correlations between leptin and FEV1or FVC ^3^ were observed and higher exhaled breath condensate leptin levels in obese and asthmatic children.^4^

Adiponectin inhibits proinflammatory cytokines, promotes vascular smooth muscle cell dilatation, and is reduced in overweight adolescents with abdominal obesity and obese children. This may be due to necrosis of adipocytes related to obesity-induced hypoxia.^2^^,^^4^ In a Brazilian study, adiponectin higher levels were observed after a multidisciplinary intervention for weight control.^5^ Madeira et al. stated that asthma in children does not appear to have a different inflammatory profile in obese versus non-obese individuals, although obese asthmatics were more symptomatic.^6^ It was proposed that high BMI and leptin, along with low adiponectin, indicate severe asthma and have an asthma-predictive value, suggesting they can be used as potential indicators for future treatment.^3^

Associations with BMI were related to *FTO* and *TMEM*18 genes. Growing evidence that the *FTO* gene would participate in childhood weight gain in African-American populations, Europe, and Brazil was observed and rs1421085 was reported to have a recessive effect on BMI.^7^ *FTO* gene was associated with BMI and asthma ^8^ and appeared downregulated in asthma.^9^ At the age of 3.5 years, *TMEM*18 rs6548238 C/C homozygotes correlated with a higher BMI-Z score, compared to the T allele.^10^

The objective of the present study was to describe possible interactions of leptin, adiponectin, *FTO* rs9939609, and *TMEM*18 rs6548238 in children and adolescents with asthma, under the influence of obesity.

## Methods

This is a cross-sectional study with children and adolescents aged 8–19 years from the Rio de Janeiro metropolitan area, Brazil, performed in a tertiary hospital, from 2017 to 2018.

Participants, selected mainly from basic health units, were included if they were children or adolescents diagnosed with asthma without overweight and obesity (below BMI-Z score +1, equivalent to BMI 25, the maximum value for normal weight, the comparison group) or with overweight and obesity {overweight [above BMI-Z score (ZIMC) +1] and obesity (above ZIMC +2), the study group]}. They were excluded if they presented other endocrine or pulmonary conditions. The study was approved by the Research Ethics Committee of the Federal University of Rio de Janeiro, process n. 798.452, CAAE 35999314.1.0000.5257. Children's parents signed the informed consent form, and participants above 12 years of age also signed the consent form. Brazilian Registry of Clinical Trials (REBEC) U1111- 1274-6649.

### Data collection

Data of participant's symptoms, triggering factors, confirmation of asthma diagnosis by a doctor, the medication used in the past year for asthma control (duration and dose), presence of rhinitis, number of asthma attacks, and number of visits to the emergency services were collected from an 8 questions questionnaire.^11^ Using these questionnaire data, the authors attempted to categorize asthma intensity by subdividing data into three parts, based on the score, namely: 0–12; 12–24, and 24–36. The higher the score, the greater the presence of asthma symptoms.

To collect anthropometric data, body weight, and height were measured to calculate the anthropometric indicators height-for-age and BMI as z scores.

Spirometry was performed using the KOKO Incorporation device, following the Brazilian Consensus on Spirometry recommendations.^12^ The correct technique, equipment calibration, and maintenance were performed during the entire procedure in order to achieve consistently accurate test results. Maximum participant effort in performing the test was necessary to avoid major errors in diagnosis. The following parameters were evaluated: forced vital capacity, FEV1, FEV1/FVC, and mean FEF_25–75_ (24). Bronchodilator tests were performed on all participants. The parameters described were evaluated before and after the participant inhaled a short-acting bronchodilator, salbutamol 100 mcg/jet, four jets per test. The bronchodilator test was considered positive when there was lung obstruction parameters reversibility, of, at least, 12 % and a 200 ml increase in FEV1.^13^

Peripheral blood was collected to measure adiponectin (Triturus equipment from Grifols was used, along with enzyme immunosorbent assay methodology) and leptin (equipment Alisei, method enzyme immunosorbent assay); studied Single nucleotide polymorphisms (SNPs) were *FTO* (rs9939609) A/T and *TMEM* (rs6548238) C/T, performed using TaqMan Master Mix Real-Time PCR assays (equipment Step One Plus PCR, Applied Biosystems, Foster City, CA).

Two groups with asthma were formed: G1 consisted of normal-weight participants, and G2 of overweight and obese participants, denominated obese. When significant data only was observed splitting the obese participants into overweight and obese, overweight is cited.

### Statistical analysis

Statistical analyses were performed using the SPSS 26.0 software package (IBM®). Means or medians, or frequencies and percentages were used to express data. Qualitative variables are presented as absolute numbers, and percentages were compared using the Chi-square test. The Pearson correlation test was used to analyze possible associations between the continuous variables. Nonparametric tests, including the Mann-Whitney test, were used for statistical qualitative and continuous variables comparisons. Confidence Interval (CI) was calculated with qualitative or continuous variables using Bootstrapping. The final significance level was 5 %.

## Results

Demographic data, spirometry variables, and laboratory tests related to nutritional classification in normal-weight and obese were described (Table 1).

**Table 1:** Demographic characteristics, pulmonary function and laboratory indices of studied patients.

CHARACTERISTICS	VARIABLES (MEAN/ MEDIAN/ IQ)	GROUP 1 (*n* = 26)	GROUP 2 (*n* = 31)	*P*
**DEMOGRAPHICS (*n*=57)**				
FEMALE (33.33 %) n (%)		9 (15.79)	10 (17.54)	0.851
AGE (years), mean, (SD)	8–19 (12.3/12/11–13.5)	12.65 (0.48)	12 (0.43)	0.280
ANTHROPOMETRY (*n* = 57)				
BMI, mean, (SD), median	15–33 (22.43/22.23/18.68–26.39)	18.76 (0.45)	25.51 (0.59)	
				
QUESTIONNAIRE (*n* = 57)				
QUESTIONNAIRE SCORE	0–36 (19.98/21/13–27)	5–32	0–36	0.516
INHALED CORTICOSTEROID USE (68 %)		19	23	0.757
				
SPIROMETRY% preview (*n* = 57)				
FEV1, mean, (SD)	28–126 % (92.3/94/84–103)	95.15 (3.37)	89.9 (3.11)	0.217
FVC, mean, (SD)	59–133 % (101/103/92–110.5)	105.19 (2.31)	98.97 (2.31)	0.072
FEV1/FVC, mean, (SD)	46–113 % (90.23/92/86–97)	90.15 (2.25)	90.29 (2.45)	0.942
FEF_25–75_, mean, (SD)	8–151 (81.53/85/65–100)	81.69 (5.72)	81.39 (5.14)	0.822
				
LABORATORY (*n* = 32)				
LEPTIN (ng/ml), mean, (SD)	0.54–42.22 12.97 /9.56 /4.98–18.71	8.70 (3.11)	16.29 (2.26)	0.003
ADIPONECTIN (µg/ml), mean, (SD)	< 0.5–15.9 (9.56/ 9.77/ 8.07- 11.47)	10.57 (1.00)	8.78 (0.69)	0.059
				
SNPS (*n* = 53)				
*TMEM* C/C (77.4 %)	41	17	24	0.567
*TMEM* T/C (18.9 %)	10	6	4	
*TMEM* T/T (3.8 %)	2	1	1	
*FTO* T/T (30.2 %)	16	5	11	0.085
*FTO* T/A (64.2 %)	34	16	18	
*FTO* A/A (5.7 %)	3	3	0	

BMI, Body mass index; FEF, Forced expiratory flow; FEV1, forced expiratory volume in 1 s; *FTO*, Fat Mass and Obesity-Associated; FVC, Forced vital capacity; IQ, Interquartile range; SD, Standard Deviation; SNPs, Single nucleotide polymorphisms; *TMEM*, Transmembrane Protein.

Age correlated directly with BMI in G1 (*p* = 0.009; *r* = 0.504, 95 % CI: 0.142–0.800) and G2 (*p* = 0.004; *r* = 0.501, 95 % CI: 0.176–0.734).

Questionnaire scores related to adiposity, age and gender. In G1, they correlated directly with age (*p* = 0.024, OR = 0.442, 95 % CI: 0.053–0.737). The highest questionnaire scores were observed in females (*p* = 0.044), mainly in the obese (*p* = 0.016).

Table 2 describes significant pulmonary function parameters related to adiposity and gender. Females using inhaled corticosteroids performed 46 %−94 % FEV1/FVC versus 94 %−106 % non-users (*p* = 0.037). Table 3 describes bronchodilation tests related to BMI. Small airway dysfunction was found in 14 (24.6 %) participants. BMI > 25 was observed mainly in non-carriers of this disease (11 × 1 carrier) (*p* = 0.002). The correlation between small airway dysfunction and bronchodilator negative test was significant since the 14 participants with the disease performed bronchodilator negative tests (*p* = 0.035; OR = 0.744, 95 % CI: 0.615–0.872).

**Table 2:** Pulmonary function parameters correlated with adiposity and gender.

Parameters	Female	Male	*p*
**Nonobese**			
FEV1/FVC (%)	86–106	52–102	0.043
FEF_25–75_ (%)	52–151	13–131	0.038
**Obese**			
FEV1 (%)	28–96	62–126	0.010
FEF_25–75_ (%)	9–109	32–131	0.036

FEV1, forced expiratory volume in 1 s; FVC, forced vital capacity; FEF, forced expiratory flow.

**Table 3:** Bronchodilation tests related to BMI and adiponectin levels.

	BD-	BD+	*p*
**Nonobese**			
BMI (Kg/m^2^)	14.55–17.16	19.4–26.84	0.049
**Obese**			
Adiponectin levels (µg/ml)	< 0.5–12.4	8.84–13	0.039

BD, Bronchodilation test.

**Figure 1:**
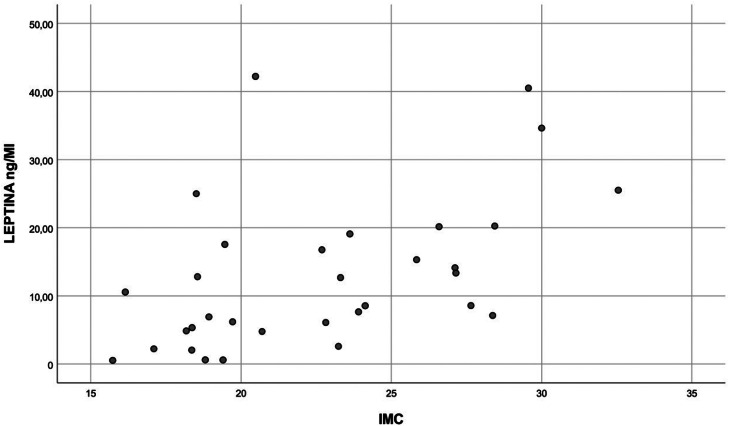
Correlation between BMI and leptin levels (*p* = 0.003).

Leptin dosages related directly with BMI: 5,34–40 ng/ml in obese x 0,54–42 ng/ml in nonobese (*p* = 0.003) (Figure 1). High leptin levels (4.78–17.55 ng/ml) correlated with female gender (*p* = 0.029), mainly in nonobese (*p* = 0.006) and small airways dysfunction non-carriers (0–42.2 ng/ml) (*p* = 0.026), compared to carriers (0–12.81 ng/ml).

When subdivided into normal-weight, overweight and obese populations, adiponectin was low in obese participants. (0–8.34 µg/ml) (*p* = 0.029). In overweight and obese participants, serum levels > 5 µg/mL correlated mainly with FEV1/FVC > 80 % [*p* = 0.035; *r* = −0.499, 95 % CI: −0.770-(−0.314)]. Table 3 describes the correlation between adiponectin levels with bronchodilation.

In the overweight and obese population, the *FTO* A allele correlated with FEV1/FVC lowest indices (46–88 %) (*p* = 0.023), was less responsive to bronchodilator tests (*p* = 0.036) and related to low adiponectin indices (0–8.84 µg/ml) (*p* = 0.021). No other significant correlations were found with *FTO. TMEM* alleles were not statistically significant when correlated with all studied parameters.

## Discussion

This study was conducted in a tertiary hospital with children and adolescents diagnosed with asthma. BMI correlated with age, older girls reported more clinical asthma complaints. Normal-weight girls presented higher FEV1/FVC and mean FEF_25–75_, while overweight and obese boys had higher FEV1 and FEF_25–75_. Girls using inhaled corticotherapy presented lower FEV1/FVC values, which indicates an obstructive condition. Negative bronchodilator tests related to lower BMI. Small airway dysfunction, described as having low FEF_25–75_ and normal FEV1, FVC and FEV1/FVC values,^14^ correlated inversely with BMI and bronchodilator tests. Serum leptin levels were lower in nonobese boys and in small airway dysfunction in overweight and obese participants. Higher levels were found in girls, obese, and in small airways dysfunction noncarriers. Adiponectin correlated inversely with obesity and directly with worse asthma. The *FTO* A allele correlated with worse spirometry results and lower adiponectin levels.

In this sample, which is not a population study, 15.8 % were overweight and 38.6 % obese. According to 2024 data from the World Obesity Federation, in Brazil, the 2020 prevalence of high BMI in children was 34 %.^15^ This fact placed obesity as a public health problem in the country.

In this work, BMI was directly related to age. In contrast, Morishita et al.^1^ in Brazil found overweight and obesity correlated inversely with age, although their sample was slightly larger than ours, and the population lived in another Brazilian city. The present results possibly reflected that older groups may choose more caloric and processed foods, some at a lower cost. Aligning with Brazilian researchers Da Silva et al.^16^ and Madeira et al.,^6^ the authors did not find differences between age, gender, and BMI.

The highest scores in the questionnaire were observed in normal-weight older females mainly the obese, but published papers are divergent on it. In the United States of America, Chen et al.^17^ found that the association between asthma history and obesity risk might be stronger among boys, as also found in a systematic review and meta-analysis in children.^18^ A publication studying the 1958 British birth cohort, a large group of 1968 girls and 2223 boys, found girls with early menarche more likely to be overweight.^19^ Although age at menarche did not explain the association between obesity and asthma in this cohort, both were independently associated with the persistence of asthma symptoms. The authors concluded that influences on body composition, unrelated to a genetic BMI predisposition, could contribute to gender differences in correlations, suggesting BMI would not be a good marker for body fat in childhood. Egan et al.^20^ conducted a systematic review of BMI and asthma in children and found overweight boys were at increased risk for asthma. When BMI was defined using Z-scores, only girls were at risk for asthma. The authors suggested that sex does not appear to be an effect modifier of childhood obesity and asthma, but various overweight/obesity measurements could influence the results, given that BMI alone cannot distinguish between fat and muscle mass. These parameters may differ by sex, since large variations in body fat distribution can occur within the same BMI percentile group. Inconsistent findings in the literature may express differences in definitions and categorizations, but could also be related to the timing of the studies in relation to other influences on body composition, such as age at menarche, which themselves may be under genetic influence, they concluded.^20^

Regarding our spirometry results, obese boys presented higher FEV1 and FEF_25–75_ rates. In a study in China, Ma et al.^2^ found FVC and FEV1/FVC in the obese group higher than in the control group. While they found FEV1 % lower than in the control group, the authors observed the opposite in obese males. This sample was twice the size of ours and participants were recruited from a hospital, whereas our sample mostly attended basic health units, which may have resulted in greater frequency/intensity of asthma. Madeira et al.^6^ observed no differences in spirometry between non-obese and obese asthmatics and no differences in the use of inhaled corticotherapy. Their sample resided in the same city as ours, with a similar *n*, but was selected from a tertiary outpatient clinic. Girls using inhaled corticotherapy, in this study, performed a worse lung function. It is possible that the indication for corticotherapy was already based on a worse clinical history, which was not revealed in the questionnaire.

The authors observed high leptin levels in girls and low in normal-weight males. A direct relationship between leptin levels and BMI was found. Al Ayed et al.^21^ showed higher leptin levels in obese asthmatic boys, but their sample did not include girls. Other works also observed higher leptin levels in obese subjects in both sexes, which did not occur in this study. Mikalsen et al.^3^ studied a sample of 384 Norwegian adolescents from a cohort selected at birth and Bodini et al.^4^ enrolled 61 Italian children and adolescents at an university hospital, a heterogeneous sample. Ma et al.^2^ advocated that BMI and leptin should be identified as risk factors for asthma in children, and if signs of obesity are present in childhood, asthma will be aggravated, along with greater risk of complications.

Adiponectin levels were lower in overweight and obese participants. Mikalsen et al.,^3^ Sparrenberger et al.^22^ and Madeira et al.^6^ also found this inverse relationship. In the Sparrenberger multi-center ERICA study of 4546 adolescents, this inverse relationship was noted especially in males. Comparison of adiponectin studies in adolescents is complex, as studies are restricted to specific populations or overweight adolescents. Also, ethnic and genetic factors seem to explain the variation found in adiponectin. In the present study, the nonobese group had high adiponectin, which was correlated with younger participants, and, in overweight and obese participants, with high FEV1/FVC and bronchodilator-positive tests. These last two findings are contradictory, as positive bronchodilator tests would indicate worse spirometry. Rodrigues et al.,^23^ in their Brazilian sample with 63 obese adolescents, as well as Mikalsen et al.,^3^ found no influence of adiponectin on spirometry. Ma et al.^2^ found that adiponectin, forced expiratory capacity in 1 s, and FEV1 % in obese subjects were lower than in the normal-weight group. Zhang et al.^24^ pointed to the presence of high leptin and low adiponectin associated with asthma diagnosis. Adipokines dosage in the present study was performed in a small sample, which limits the assessments.

In the obese group, *FTO* allele A correlated with low FEV1/FVC, bronchodilator negative tests, and low adiponectin. The authors found no associations between BMI and *FTO* rs9939609 or *TMEM*18 rs6548238. Srivastava,^25^ Zhang,^24^ Melén,^26^ Lourenço,^27^ Granell,^28^ Loos ^29^ and their colleagues studied the *FTO* gene, and all found it related to a higher risk of obesity. Krishnan et al.^30^ found associations in a sample of New Zealand European children. Loos et al.^29^ noted that minor allele increases BMI by 0.39 kg/m 2 (or 1.130 g in body weight), and the risk of obesity by 1.20-fold, occurring across diverse age groups and ancestry, with the largest effect in young adulthood. Melén et al.^8^ found *FTO* associated with BMI and asthma and stated that this gene is important for childhood BMI, regardless of asthma status.^26^ Granell et al.^28^ concluded that the effects of BMI-related SNPs on asthma are mediated by their effects on BMI, but possibly also by genetic pleiotropy, as studies on twins suggested that a proportion of the covariation between obesity and asthma is explained by shared genetic factors. Genome-wide linkage studies have identified overlapping regions of the genome associated with both asthma and obesity, but no genetic variant associated with obesity and asthma has been consistently identified. The authors argued that some of the heritability is explained by non-coding variation in Deoxyribonucleic acid, such as methylation or other epigenetic effects. Lourenço et al.^27^ suggested that *FTO* affects adiposity in an age-dependent manner in children and that nutritional factors may modify genotypic effects. This gene has been associated with lower BMI in the first years of life and earlier adiposity recovery, followed by a subsequent greater gain in BMI from late infancy. Zhang et al.^24^ pointed out that this association may be modified by dietary characteristics, particularly the distribution of fatty acids and lower protein intake by children. Loos et al.^29^ agreed with Zhang et al.^24^ on the influence of this gene on subtle changes in food intake and preferences on the regulation of translation and growth. Because *FTO* is expressed in the brain, where amino acid sensing can influence the activity of pathways that control food intake, this gene can lead to the consumption of more food, as well as alter nutrient preference, possibly suggesting that *FTO* status can influence dietary macronutrient composition. Thus, the role of *FTO* in amino acid sensing may provide some clues toward understanding the cellular basis of this physiological phenomenon and reveal new therapeutic targets in the battle against the growing prevalence of obesity and asthma.

The present study has several limitations, mainly due to the small sample size, which was further reduced when the determination of adipokines was performed, precluding the use of linear regression. The participants were largely from a third-world population, with lower socio-economic backgrounds, limited access to health services, and fewer financial government resources for asthma treatment. In addition, this poverty of resources is often consistent with challenging household conditions, exposing this population to more airborne fungi and house dust mite allergen concentrations.

As the Brazilian population is strongly characterized by racial mixture, studies addressing different population groups are needed to better understand the correlation of genetic polymorphisms with asthma and obesity. The information generated by this work about the possible relations of adipokines and genetic polymorphisms on the relationship between asthma and obesity may contribute to the understanding of asthma pathophysiology, under the influence of obesity.

In conclusion, this study found, in the whole population, that BMI correlated directly with age and leptin levels. In nonobese participants, the older ones presented more asthma complaints and high adiponectin levels. In obese, adiponectin levels were low, females complained more of asthma and boys performed higher FEV1, FEV1/FVC, and positive to bronchodilation. More studies are necessary with larger-scale and longitudinal design cohorts, for better elucidation of the genetic polymorphisms and adipokines roles in the pathophysiology of asthma and obesity, two diseases so frequent in the world today.

## Conflicts of interest

The authors declare no conflicts of interest.
